# Influence of Multifrequency Ultrasound-Assisted Freezing on the Flavour Attributes and Myofibrillar Protein Characteristics of Cultured Large Yellow Croaker (*Larimichthys crocea*)

**DOI:** 10.3389/fnut.2021.779546

**Published:** 2021-12-15

**Authors:** Xuan Ma, Dazhang Yang, Weiqiang Qiu, Jun Mei, Jing Xie

**Affiliations:** ^1^College of Food Science and Technology, Shanghai Ocean University, Shanghai, China; ^2^National Experimental Teaching Demonstration Center for Food Science and Engineering Shanghai Ocean University, Shanghai, China; ^3^Shanghai Engineering Research Center of Aquatic Product Processing and Preservation, Shanghai, China; ^4^Shanghai Professional Technology Service Platform on Cold Chain Equipment Performance and Energy Saving Evaluation, Shanghai, China

**Keywords:** large yellow croaker, multi-frequency ultrasound assisted freezing, microstructure, flavour attributes, myofibrillar protein characteristics

## Abstract

The influence of multifrequency ultrasound-assisted freezing (UAF) as compared with single- and dual-UAF on the flavour, microstructure, and myofibrillar proteins (MPs) of cultured large yellow croaker was investigated to improve food quality in a sustainable way and address the major global challenges concerning food and nutrition security in the (near) future. Multifrequency UAF-treated samples had lower total volatile basic nitrogen values during freezing than single- and dual-UAF-treated samples. Thirty-six volatile compounds were identified by solid-phase microextraction (SPME) coupled to gas chromatography–mass spectrometry (GC-MS) during freezing, and the multifrequency UAF-treated samples showed significant decreases in the relative contents of fishy flavoured compounds, including 1-penten-3-ol and 1-octen-3-ol. In addition, multifrequency UAF treatment better maintained a well-organised protein secondary structure by maintaining higher α-helical and β-sheet contents and stabilising the tertiary structure. Scanning electron microscopy images indicated that the ice crystals developed by the multifrequency UAF were fine and uniformly distributed, resulting in less damage to the frozen large yellow croaker samples. Therefore, multifrequency UAF improved the flavour attributes and MP characteristics of the large yellow croaker samples. Overall, multifrequency UAF can serve as an efficient way for improving food quality and nutritional profile in a sustainable way.

## Introduction

Large yellow croaker (*Larimichthys crocea*) is an important marine cultured fish that is widely distributed in China. Freezing is an efficient method to extend the shelf life of large yellow croaker by controlling microbial growth and decreasing biochemical reactions (1). The quality of frozen fish is affected by the size of the ice crystals and their location within the fish (2). The size and homogeneity of ice crystals are largely affected by the freezing rate. Generally, rapid freezing can produce fine and uniform extra- and intracellular ice crystals that cause less damage to food structures. In contrast, slow freezing generates large and uneven extracellular ice crystals, which can damage the muscle structure and decrease the quality of the food (3). Several innovative and emerging freezing techniques have been used to provide a promising means to optimise the crystallisation of frozen fish. These techniques, including radio frequency-assisted freezing (4), high-pressure freezing (5), and ultrasound-assisted freezing (UAF) (6–11) can change the nucleation, nucleation rate, and crystal growth of food substrates during freezing.

Ultrasound-assisted freezing has a favourable effect on the process of food freezing due to its cavitation effects (12, 13). The movement of cavitation bubbles can lead to a microstreaming effect, thus improving the efficiency of heat and mass transfer (14). Furthermore, cavitation bubbles can serve as crystal nuclei and increase the number of nucleation sites. Large ice crystals can be fragmented by microstreaming, resulting in a further decrease in the size of ice crystals (15). Some studies have indicated that ultrasound facilitates the freezing processes of different food materials. Sun et al. (16) evaluated the effects of UAF with different ultrasound powers on myofibrillar protein (MP) structures and the thermal stability of common carp and found that ultrasound treatment at 175 W significantly reduced the changes in protein structure and preserved the thermal stability of the protein during freezing. Single-frequency ultrasounds have been frequently used in the freezing investigations mentioned above. The frequency is closely associated with the intensity of ultrasonic cavitation and is an important indicator of the acoustic-chemical reaction (17). It also has an impact on the bubble activity induced by the bubble rupture, which has an impact on the product quality. The coupling of ultrasonic energy peaks when the natural resonant frequency of the bubble is commensurate with the ultrasonic frequency (18). Compared to single-frequency ultrasound, multifrequency ultrasound has been indicated to greatly improve cavitation effects (19). Ma et al. (20) proposed that the effect of dual-frequency flat ultrasound is more significant than that of single-frequency flat ultrasound because of its homogeneous energy dissipation over a broader region. The efficiency of various acoustic and cavitation devices, such as dual frequency and single frequency devices, was investigated by Jawale et al. (21). They pointed out that dual-frequency acoustic-chemical reactors show more effective performance during use than single-frequency acoustic-chemical reactors. Wang et al. (22) investigated the influence of multifrequency ultrasound-assisted thawing on the structure and rheological characteristics of MPs in small yellow croaker and found that dual-frequency ultrasonic thawing could effectively minimise the changes in structure and preserve the rheological characteristics of MPs compared with single-frequency ultrasonic thawing.

We are currently and will continue to face major global challenges concerning food and nutrition security. It is expected that stresses to food systems continue to appear in the (near) future, for example, pandemics, economic shocks, and climate change effects at local and global scales. As previously stated, much progress has been made regarding the ultrasound-assisted immersion freezing of food materials, since it was first reported ~20 years ago (23). However, many studies on freezing fish products by ultrasound remain in their infancy, and the majority still adopt single-frequency ultrasound for freezing. To the best of our knowledge, there are few studies on the application of multifrequency UAF in food materials. Considering these backgrounds, this study was designed to disseminate solutions based on multifrequency UAF, an innovative and emerging non-thermal food engineering technology, to build sustainable and resilient food systems, improve nutritional profile of foods, and increase food and nutrition security. This study evaluated the changes in total volatile basic nitrogen (TVB-N), scanning electron microscopy (SEM) observations, flavour attributes, free amino acids (FAAs), and MP characteristics of large yellow croaker samples frozen by multifrequency ultrasound (consisting of single-, dual-, and triple-frequency ultrasound).

## Materials and Methods

### Sample Preparation

Fresh large yellow croakers (weighing 550 ± 25 g) were purchased from a local market in Luchao Port town (Shanghai, China). The fresh fish were prepared by a local processor, and the viscera and gills were removed. Then, the treated fish were transported to our laboratory on ice within 30 min and washed with saline solution containing 0.9% sodium chloride.

### Freezing Process

**Figure 1:**
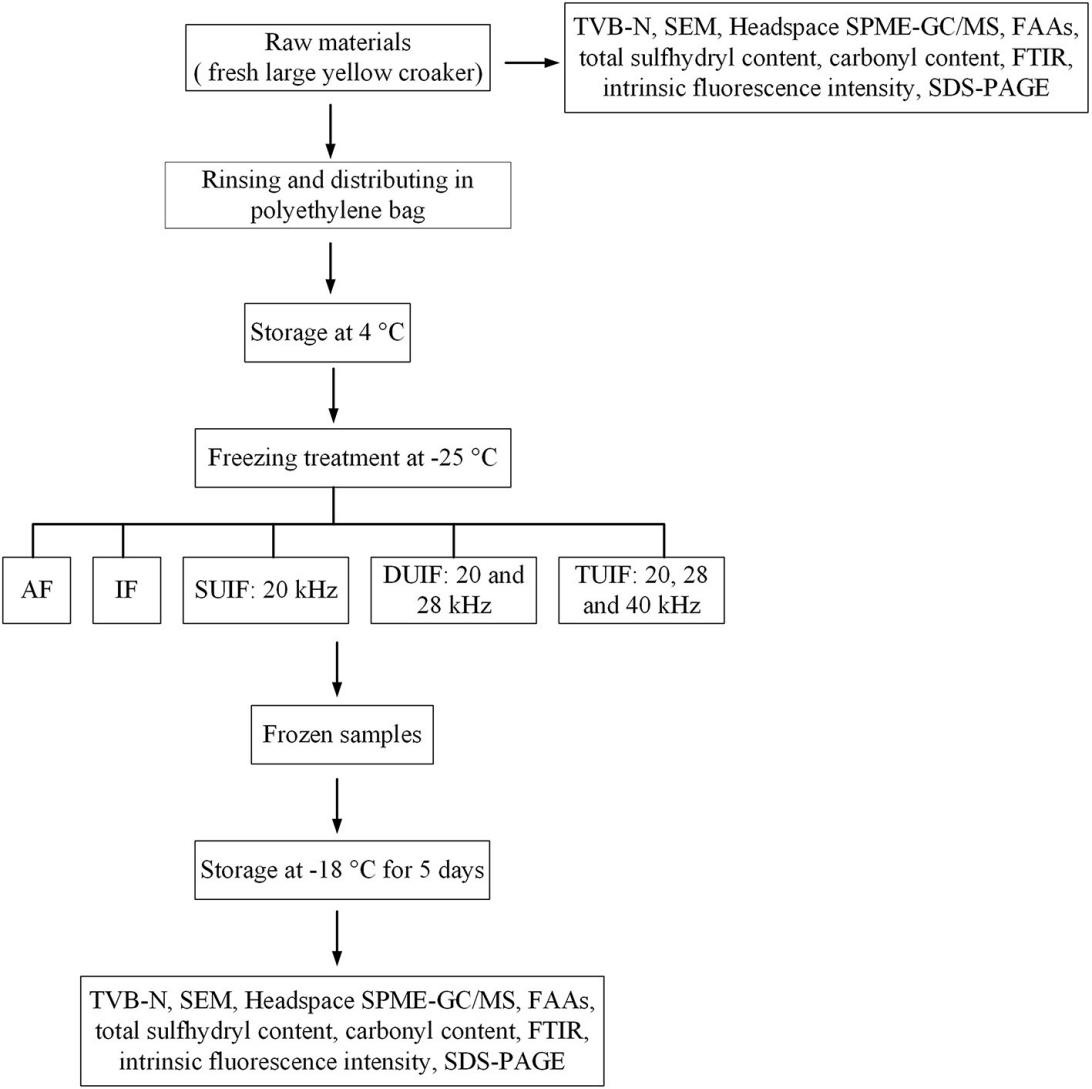
Flow diagram of experimental step.

**Figure 2:**
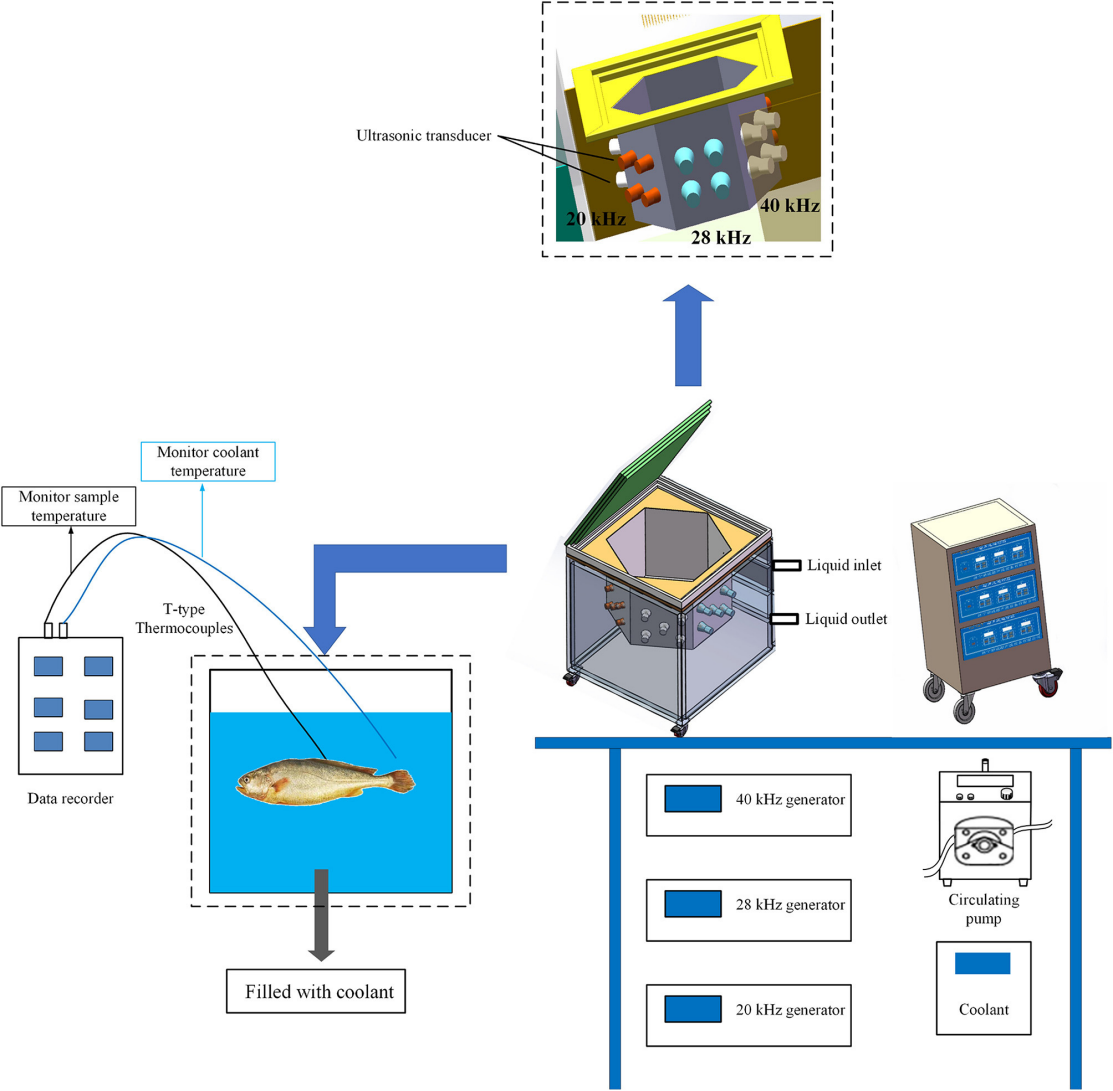
The diagram of the multifrequency ultrasound-assisted immersion freezing unit.

According to the experimental procedure of Ma et al. (24), the fish samples were subjected to the following five different freezing treatments: air freezing (AF), immersed freezing (IF), and ultrasound-assisted immersed freezing (UIF) linked with single frequency at 20 kHz (SUIF), UIF linked with dual frequency at 20/28 kHz (DUIF), and ultrasound-assisted immersed freezing linked with triple frequency at 20/28/40 kHz (TUIF). The multifrequency ultrasound-assisted freezer was customised by Xiecheng Ultrasonic Equipment Co., Ltd. (Jining, Shandong, China). This system used 29.3% calcium chloride brine solution (w/v) as the coolant. The temperature of coolant was set to (−25.0 ± 0.5)°C with a cryogenic circulation pump. A detailed flow diagram of the experimental procedure is presented in Figure 1. A diagram of the multifrequency ultrasound-assisted immersion freezing unit is presented in Figure 2.

### Evaluation of TVB-N

Total volatile basic nitrogen values were examined with a Kjeldahl apparatus (Kjeltec8400, Foss, Hilleroed, Denmark). The results of TVB-N are shown as mg N/100 g of large yellow croaker samples (25).

### SEM Observation

The morphology of frozen dorsal muscle samples was observed by SEM according to the procedure proposed by Xu et al. (17) with minor modifications. The freeze-dried large yellow croaker samples were placed on one surface of a two-sided adhesive tape and coated with a thin gold layer; then the morphology of the fish samples was observed by extreme-resolution analytical field emission SEM (Mira 3 XH, Tescan, Brno, Czech Republic). For the SEM observation, the acceleration voltage was 5 kV.

### Analysis of Headspace by Solid Phase Microextraction and Gas Chromatography–Mass Spectrometry

Volatile organic compounds (VOCs) of samples under different freezing conditions were separated and detected using the procedure proposed by Li et al. (26). A 2-g mashed large yellow croaker sample and 5 ml of 25% NaH_2_PO_4_ solution were mixed and placed in a 20 ml headspace vial. The VOCs were characterised *via* comparison with commercial reference compounds obtained from Sigma–Aldrich (St. Louis, MO, USA), and their mass spectra were compared with those included in the NIST 2011.

### Analysis of FAAs

Free amino acids were determined using the method proposed by Yu et al. (27). A 2-g chopped large yellow croaker sample and 10 ml of 5% cold trichloroacetic acid were mixed and homogenised at 10,000 × g for 10 min. The extraction and centrifugation were repeated and the combined supernatants were diluted to 25 ml. The supernatant was filtered through a 0.22 μm filter. An automatic amino acid analyser (Hitachi L-8800, Tokyo, Japan) was used for evaluation.

### Extraction of MP

Myofibrillar protein was obtained as proposed by Guo et al. (28) with minor modifications. In short, 2 g of minced large yellow croaker flesh was mixed with 20 ml of prechilled Tris-buffer A (0.05 M KCl, 20 mM Tris-maleate, pH 7.0) and homogenised in an ice bath for 30 s. The mixture was then centrifuged at 10,000 r/min and 4°C for 15 min and the precipitate was retained. The above homogenisation and centrifugation steps were repeated twice, and the precipitate was mixed with 20 ml of prechilled Tris-buffer B (0.6 M NaCl, 20 mM Tris-maleate, pH 7.0). After that, the mixture was homogenised in an ice bath for 30 s and left for 3 h. The supernatant was obtained as a myofibril protein solution by centrifugation at 10,000 r/min and 4°C for 15 min. The solutions were kept frozen at −40°C until further analysis.

### MP Primary Structure

#### Determination of Total Sulfhydryl Content

The total sulfhydryl content was measured by the procedure proposed by Li et al. (29) with slight modifications. A 1 g dorsal muscle large yellow croaker flesh sample was homogenised in 10 ml of an 8 M urea solution mixed with 0.6 M NaCl. Afterwards, the mixture was centrifuged at 2,500 g/min at 4°C for 10 min. Then, 0.5 mL of the supernatant was combined with 4.5 ml of buffer A (8 M urea, 3 mM EDTA, 1% SDS, 0.2 M Tris-HCl, pH 8.0) and mixed homogeneously. Subsequently, 0.625 mL of buffer B (10 mM DTNB, 10 mM Tris-HCl, pH 8.0) was supplemented, and the mixture was maintained at 40°C for 20 min. The absorbance was measured at a wavelength of 405 nm at room temperature, and colorimetry was completed within 30 min. The final calculated value of total sulfhydryl content is given as μmol/g protein.

#### Determination of Carbonyl Content

The carbonyl group content was evaluated using derivatisation with 2,4-dinitrophenyl hydrazine according to the method described by Zhang et al. (30). The carbonyl groups reacted with 2,4-dinitrophenylhydrazine to produce protein hydrazone. The results are given as nmol/mg protein.

### MP Secondary Structure

The secondary structure of the MP solution was determined by Fourier transform infrared (FTIR) spectroscopy. The dried myofibril sample was ground with potassium bromide powder to form a uniform powder. Then, it was pressed into a sheet, and the secondary structure was measured using a Fourier infrared spectrometer (Nicolet IS50; Thermo Scientific Inc., Waltham, MA, USA). The test parameters were as follows: the scanning wavelength range was 4,500–500 cm^−1^, the resolution was 4 cm^−1^, and the number of scans was 32 (31).

### MP Tertiary Structure

The intrinsic fluorescence intensity (IFI) of extracted myofibril samples was measured using an AFS-9230 fluorescence spectrophotometer (Beijing Titan Instruments Co., Ltd., China). The acquisition parameters are shown below. The emission spectra range was 300–400 nm and the scanning speed was 1,200 nm/min. The maximum fluorescence wavelength (λmax) of the fluorescence emission spectrum was documented (29).

### Statistical Analysis

SPSS 22.0 software was used to statistically evaluate the experimental results which are reported as the means ± standard deviation. Significant differences (*p* < 0.05) in the mean values of treatments were determined using the one-way ANOVA and Duncan's multiple range tests.

## Results and Discussion

### Total Volatile Basic Nitrogen

**Figure 3:**
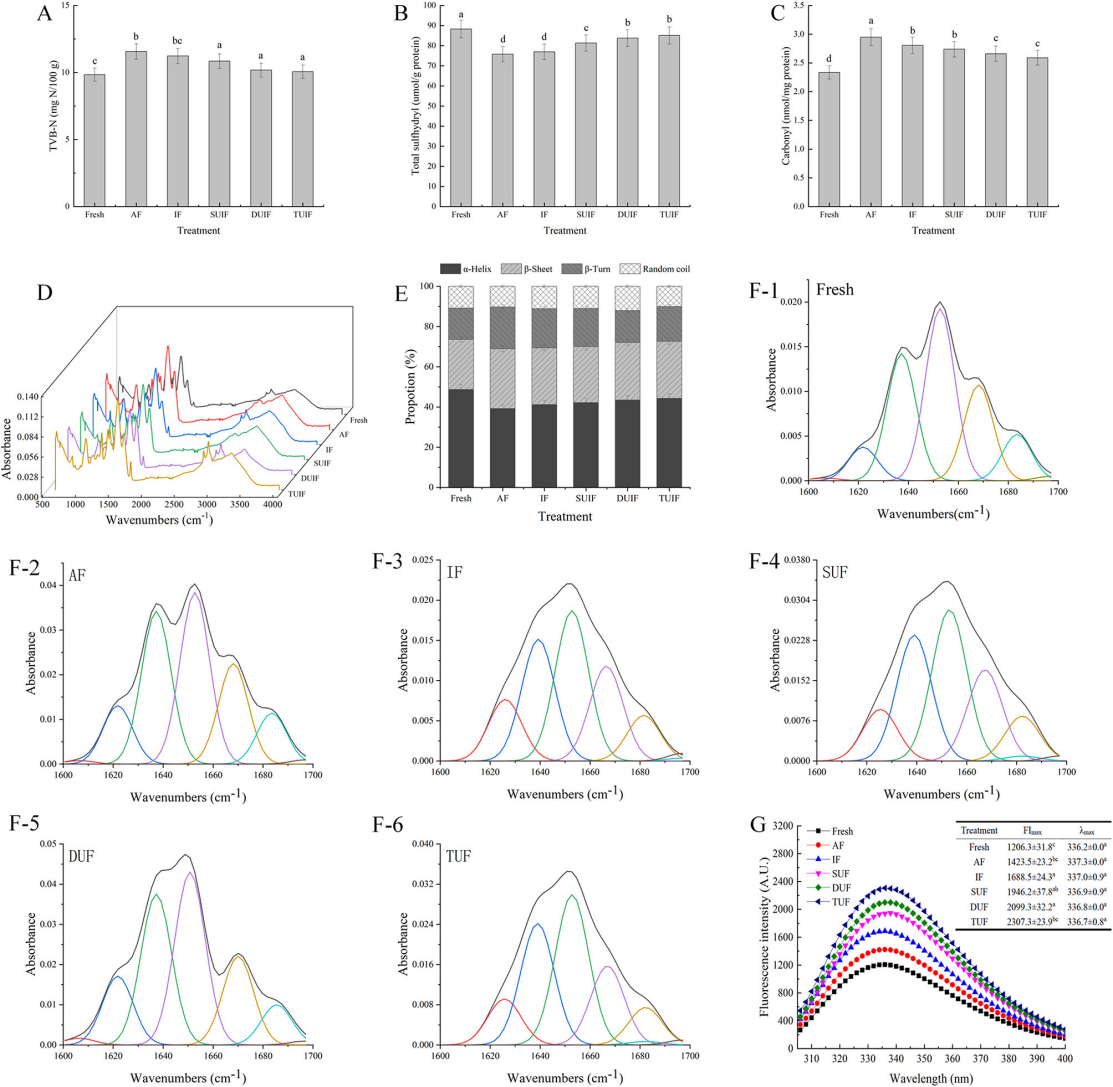
Changes in total volatile basic nitrogen values (TVB-N) **(A)**, total sulfhydryl groups **(B)**, carbonyl groups **(C)**, Fourier transform infrared spectroscopy (FTIR) **(D)**, secondary structure content **(E)**, second-derivative-fitted curve of the amide I band **(F1–F6)** and intrinsic fluorescence intensity (IFI) **(G)** of MP of large yellow croaker under different freezing treatments.

Total volatile basic nitrogen quantifies alkaline volatile compounds, including ammonia, trimethylamine, methylamine, dimethylamine, and so on, which is the primary parameter for evaluating the variation in freshness of aquatic products (32). The TVB-N content of the fresh large yellow croaker samples was 9.84 mg N/100 g (Figure 3A). The TVB-N values in the AF- and IF-treated samples were higher than those in the UAF sample. AF and IF treatment can form large ice crystals through slow freezing to puncture the cells, increase the amount of water in the extracellular space, and cause nutrient leakage, which provides a suitable culture environment for microorganisms and results in an increase in TVB-N in fish (33, 34). A significant difference among the samples with UIF treatments was not observed (*p* > 0.05), and the lowest TVB-N values were found in the TUIF-treated samples (10.85, 10.18, and 10.06 mg N/100 g for the SUIF, DUIF, and TUIF samples, respectively). The possible reason behind this is that the higher operating frequency of triple-frequency ultrasound overcomes the disadvantages of single-frequency and dual-frequency ultrasound, such as non-uniform energy consumption per unit volume and directional sensitivity. In addition, triple-frequency ultrasound can produce more cavitation bubbles and resonances named “combination resonances,” as well as greater sonochemical energy (35). The TVB-N values of the TUIF samples were reduced by 12.98 and 10.50% compared with that of the IF and AF samples, respectively, suggesting that rapid freezing can diminish the generation of TVB-N in fish samples. Sun et al. (36) also stated that the TVB-N of common carp samples after UAF treatments was less than that of samples after AF and IF treatment.

### Scanning Electron Microscopy

**Figure 4:**
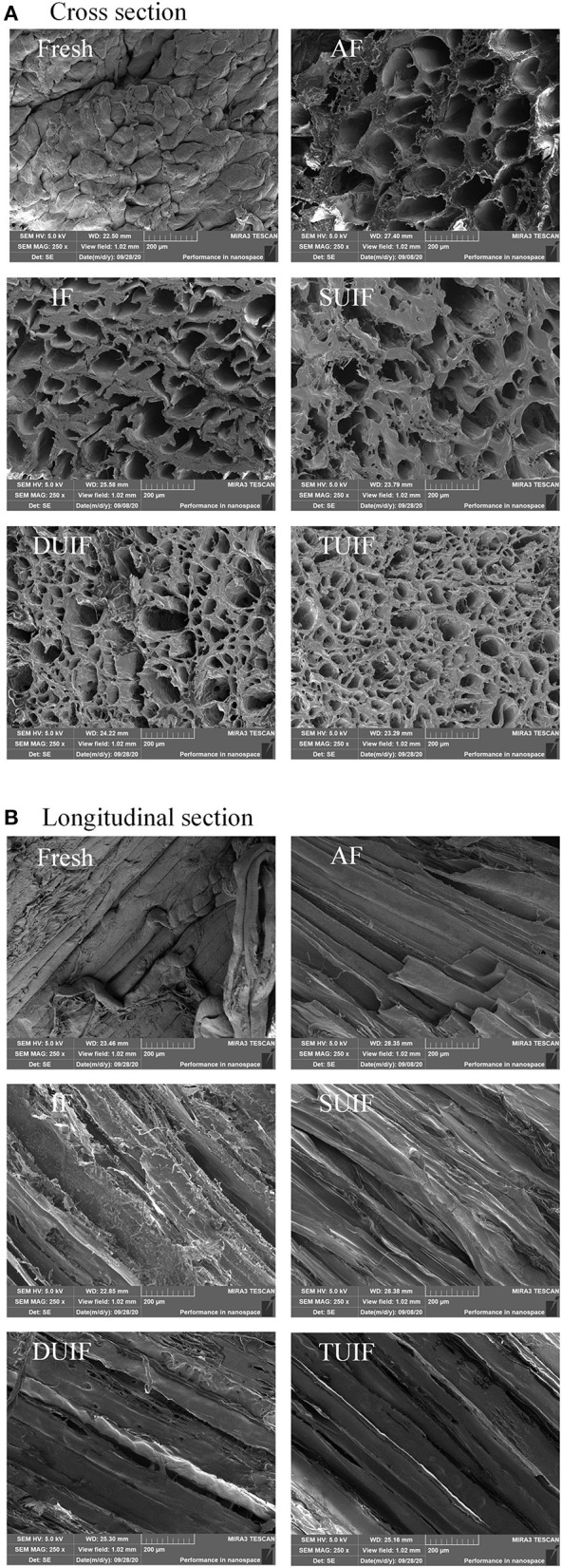
Changes in SEM cross section **(A)** and longitudinal section **(B)** of large yellow croaker muscles microstructure after different freezing treatments (magnification: 250).

The SEM images of large yellow croaker muscle (cross-section and longitudinal section) under different freezing treatments are shown in Figures 4A,B. The pores in the SEM images are connected with the location and size of ice crystals. The largest pore size was observed for the AF-treated samples compared with the other samples. The microstructure of large yellow croaker frozen by UIF was better than that of the samples frozen under AF and IF. The microstructure of TUIF samples was found to be the most compact and had the smallest holes, indicating that TUIF treatment resulted in the smallest and most uniformly distributed ice crystals among the fish samples. The multifrequency ultrasonic treatments resulted in a reduction in the size of ice crystals developed during the freezing of samples. The mechanism for small ice crystal formation can be roughly divided into three points. (i) A higher freezing rate and shorter phase transition time can contribute to the rapid conversion of freezable moisture into ice crystals (11, 37); (ii) large dendritic ice crystals can be broken into small fragments, consequently enhancing secondary nucleation (38, 39); and (iii) cavitation bubbles can be applied as crystal nuclei once they attain the critical size of ice nucleation, leading to an increase in nucleation sites (10, 15). Zhu et al. (40) also observed that the ice crystals generated by the TUIF and DUIF treatments were finer and more homogeneously distributed on potato samples. It has been reported that multifrequency ultrasound can significantly enhance the cavitation effect in contrast to single-frequency ultrasound (41). Jin et al. (42) stated that multifrequency ultrasound pretreatment can enhance the enzymolysis of corn gluten meal under various enzymolysis conditions. Nevertheless, the mechanism of action of multifrequency ultrasound has not been intensively investigated. The potential mechanisms are primarily the reinforcement of mechanical perturbations and the augmentation of cavitation nuclei associated with multifrequency ultrasound (41).

### Volatile Compounds

In total, thirty-six major VOCs were characterised from the large yellow croaker samples throughout for the whole freezing process, without considering aromatic compounds, hydrocarbons, and amine components. As presented in Table 1, these VOCs included alcohols, aldehydes, ketones, esters, and other compounds.

**Table 1:** Retention index and area of main volatile compounds identified in large yellow croaker under different freezing treatment.

**Name**	**Treatments**						
	**RI**	**Fresh**	**AF**	**IF**	**SUIF**	**DUIF**	**TUIF**
**Alcohols**							
1-Octanol	1,560.6	ND	1.83 × 10^5^	ND	ND	1.24 × 10^5^	ND
1,4-Butanediol	1,926.2	1.03 × 10^6^	4.08 × 10^5^	1.30 × 10^5^	6.69 × 10^4^	3.55 × 10^5^	ND
Ethanol	930.16	7.91 × 10^5^	1.20 × 10^6^	8.38 × 10^6^	8.37 × 10^6^	7.53 × 10^6^	9.96 × 10^6^
3-Methyl-2-butanol	1,123.7	9.57 × 10^5^	3.13 × 10^5^	5.57 × 10^4^	4.01 × 10^5^	ND	ND
2-Decanol	1,143.1	4.82 × 10^5^	2.65 × 10^4^	2.98 × 10^5^	3.51 × 10^5^	ND	4.34 × 10^5^
1-Butanol	1,164.7	2.14 × 10^6^	6.20 × 10^6^	ND	ND	2.52 × 10^6^	1.22 × 10^5^
1-Penten-3-ol	1,173.5	2.39 × 10^6^	3.65 × 10^6^	3.37 × 10^6^	3.16 × 10^6^	3.05 × 10^6^	2.70 × 10^6^
3-Methyl-1-butanol	1,218.5	1.87 × 10^6^	5.96 × 10^6^	ND	ND	ND	ND
2-Methyl-1-butanol	1,216.6	1.07 × 10^5^	ND	ND	ND	ND	ND
1-Pentanol	1,258.2	5.58 × 10^5^	1.50 × 10^6^	4.29 × 10^5^	5.03 × 10^5^	6.17 × 10^5^	4.23 × 10^5^
2-n-Propyl-1-heptanol	1,283.1	6.34 × 10^5^	1.88 × 10^6^	3.58 × 10^5^	5.59 × 10^5^	4.53 × 10^6^	8.07 × 10^5^
1-Hexanol	1,356.5	4.60 × 10^5^	1.40 × 10^7^	5.65 × 10^5^	3.23 × 10^6^	ND	5.16 × 10^5^
1-Octen-3-ol	1,450	1.27 × 10^6^	8.60 × 10^6^	1.68 × 10^6^	1.45 × 10^6^	1.45 × 10^6^	1.37 × 10^6^
2-Ethyl-1-hexanol	1,490.2	3.96 × 10^5^	5.22 × 10^6^	2.18 × 10^6^	7.10 × 10^5^	8.79 × 10^5^	8.06 × 10^5^
**Aldehydes**							
Propanal	852.24	5.37 × 10^6^	1.25 × 10^5^	1.04 × 10^7^	6.65 × 10^7^	1.16 × 10^7^	6.85 × 10^5^
Butanal	885.36	4.65 × 10^4^	1.22 × 10^5^	1.51 × 10^5^	9.14 × 10^4^	7.86 × 10^4^	6.28 × 10^4^
2-Methyl-butanal	908.29	7.57 × 10^5^	1.60 × 10^5^	ND	1.86 × 10^5^	ND	1.55 × 10^5^
3-Methyl-butanal	911.27	1.81 × 10^6^	1.42 × 10^6^	ND	1.69 × 10^5^	4.37 × 10^4^	1.77 × 10^5^
Pentanal	969.23	1.25 × 10^6^	1.40 × 10^7^	1.17 × 10^6^	1.34 × 10^6^	1.16 × 10^6^	9.71 × 10^5^
Hexanal	1,076	1.16 × 10^7^	8.67 × 10^5^	9.19 × 10^6^	1.06 × 10^7^	9.35 × 10^6^	7.65 × 10^6^
Heptanal	1,172	5.65 × 10^5^	ND	ND	3.16 × 10^6^	4.55 × 10^6^	2.70 × 10^6^
Octanal	1,277.6	5.48 × 10^5^	1.84 × 10^6^	ND	5.08 × 10^5^	5.34 × 10^5^	6.26 × 10^5^
Nonanal	1,384.6	9.64 × 10^5^	ND	9.73 × 10^5^	1.40 × 10^6^	1.17 × 10^6^	1.13 × 10^6^
(E, E)-2,4-Heptadienal	1,485.9	3.96 × 10^5^	2.37 × 10^6^	ND	ND	ND	ND
Benzaldehyde	1,511.4	3.31 × 10^7^	ND	ND	1.10 × 10^6^	1.87 × 10^6^	2.49 × 10^6^
**Ketones**							
Acetone	859.89	2.68 × 10^4^	ND	3.81 × 10^4^	3.46 × 10^4^	3.82 × 10^4^	3.39 × 10^4^
Methyl Isobutyl Ketone	1,003.7	1.96 × 10^5^	7.71 × 10^6^	ND	ND	2.09 × 10^5^	1.26 × 10^5^
2,3-Pentanedione	1,055.2	2.04 × 10^5^	6.99 × 10^6^	5.72 × 10^6^	4.53 × 10^6^	3.97 × 10^6^	3.18 × 10^6^
1-(p-Tolyl) butan-1-one	1,933.1	1.27 × 10^6^	ND	8.11 × 10^4^	8.25 × 10^5^	4.73 × 10^7^	4.48 × 10^5^
2-Butanone	899.58	ND	ND	ND	2.89 × 10^5^	6.07 × 10^5^	7.22 × 10^5^
3-hydroxy-2-butanone	1,283.4	ND	6.80 × 10^5^	1.45 × 10^6^	1.06 × 10^6^	1.31 × 10^6^	6.52 × 10^5^
**Esters**							
Ethyl Acetate	891.73	1.50 × 10^5^	4.89 × 10^6^	ND	5.32 × 10^5^	2.81 × 10^5^	5.64 × 10^5^
n-Caproic acid vinyl ester	1,315.9	2.69 × 10^6^	ND	ND	2.10 × 10^6^	2.25 × 10^6^	ND
Monolaurin	1,835.7	ND	1.78 × 10^5^	ND	ND	1.79 × 10^5^	ND
Methyl acetoacetate	1,123.7	ND	ND	1.87 × 10^6^	3.63 × 10^4^	6.89 × 10^5^	ND
**Others**							
2-Ethylfuran	941.63	3.52 × 10^6^	2.77 × 10^6^	3.31 × 10^6^	4.20 × 10^6^	3.64 × 10^6^	3.22 × 10^6^

*ND, not detected*.

Fourteen alcohol compounds were characterised, and the levels of 1-octen-3-ol and 2-ethyl-1-hexanol showed a certain increase during the freezing process. 1-penten-3-ol, ethanol, 1-pentanol, 2-*n*-propyl-1-heptanol, 1-octen-3-ol, and 2-ethyl-1-hexanol exhibited relatively high abundance during the whole freezing process of the large yellow croaker samples. Among them, 1-penten-3-ol is regarded as a highly essential volatile substance possessing green, vegetal, and burnt flavour descriptives, which has been suggested to be an indicator of lipid oxidation in chilled Atlantic horse mackerel meat (43). Furthermore, 1-octen-3-ol is a significant provider of off-flavours because of its low odour threshold. 1-Penten-3-ol and 1-octen-3-ol in the samples with UIF treatment exhibited lower abundances than those in the samples with AF and IF treatment. The levels of 1-penten-3-ol in SUIF-, DUIF-, and TUIF-treated samples were reduced by 13.42, 16.44, and 26.03% compared with AF-treated samples, respectively, and by 6.23, 9.50, and 19.88% compared with IF-treated samples, respectively. The levels of 1-octen-3-ol in the SUIF-, DUIF-, and TUIF-treated samples decreased by 83.14, 83.14, and 84.07% compared with AF-treated samples, respectively, and by 13.69, 13.69, and 18.45% compared with IF-treated samples, respectively. In particular, the levels of 1-penten-3-ol and 1-octen-3-ol in the TUIF-treated sample were similar to those in the fresh samples. The triple-frequency ultrasound can produce obviously higher improvement in cavitation yield in comparison with the single-frequency and dual-frequency ultrasound. In addition, the triple-frequency ultrasound showed more combination resonances, which can generate a much broader range of bubble sizes compared with the single-frequency and dual-frequency ultrasound (44). TUIF treatment generated small and homogeneously distributed ice crystals in the muscle tissue *via* a comparatively quick-freezing rate, which better protected the cell properties and caused the muscles to be of great quality. In addition, UIF efficiently inhibited both the action of 15-lipoxygenase on EPA (20:5n-3) and of 12-lipoxygenase on arachidonic acid (45).

Aldehyde compounds are principally derived from the oxidation of polyunsaturated fatty acids. Generally, aldehydes containing three to four carbon atoms generate strongly irritating odours, while those containing five to nine carbon atoms generate a lipidic aroma. In some cases, the Strecker degradation of amino acids can produce aldehydes. For instance, benzaldehyde is obtained through the degradation of phenylglycine, as a pleasant fruity almond-like aroma in large yellow croaker (46). Benzaldehyde showed higher relative concentrations in the fresh samples, which is in accordance with the study performed by Tan et al. (47). However, benzaldehyde was not observed in the AF and IF samples, which could be due to a greater degree of lipid oxidation as a result of severe damage to the muscle structure. Benzaldehyde, as well as octanal and heptanal, have been characterised as components of the aroma of fresh fish (48). The occurrence of 2,4-heptadienal may be attributed to the autoxidation of eicosapentaenoic acid (20: 5ω-3) (EPA) (49).

Ketone compounds were found at relatively low levels and exerted less of an effect on the flavour than aldehydes. Among ketones, increases in 3-hydroxy-2-butanone and 2,3-pentanedione levels occurred, the latter serving as an index of lipid oxidation in the muscles of frozen fish. 2,3-Pentanedione exhibited a lower abundance in the UIF-treated samples than in the AF- and IF-treated samples. The levels of 2,3-pentanedione in the SUIF-, DUIF-, and TUIF-treated samples were reduced by 35.19, 43.20, and 54.51% compared with the AF-treated samples, respectively, and by 20.80, 30.59, and 44.41% compared with the IF-treated samples, respectively. Specially, the levels of 2,3-pentanedione in the TUIF-treated sample (3.18 × 10^6^) were nearly the same as those in the fresh samples. TUIF treatment better protected the cell structure, thereby improving muscle quality. In addition, UIF efficiently inhibited lipid oxidation by decreasing the activities of lipase, phospholipase, and lipoxygenase (50). 3-Hydroxy-2-butanone was detected only after freezing treatment and is regarded as a potential indicator of marine fish. The formation of 3-hydroxy-2-butanone is mainly associated with lactic acid bacteria (51). VOCs derived from lipid autoxidation are recognised as the main factors for the quality deterioration of fish. In terms of the changes in VOCs, the peak areas of the off-odour compounds in samples were lower after UIF treatment, indicating that UAF inhibited lipid oxidation.

### FAAs Analysis

Free amino acids are essential factors involved in the flavour development of aquatic products. The taste properties of FAAs are dependent on the structures of the functional groups and side chain R groups. Most D-amino acids are mainly sweet; Met, Gly, Thr, Ala, and Ser with short side chains are mainly sweet and umami; and Tyr, Phe, Ile, Val, and Leu with large and long side chains are mainly bitter (52). Significant differences (*p* < 0.05) were observed in the total FAA contents among all samples during the freezing process. The total FAA levels in the UAF and IF samples were notably higher than those in the AF samples. The increase in FAA content is a consequence of the decomposition of proteins and peptides caused by proteolytic enzymes, while its reduction is a consequence of the interaction of those amino acids with other compounds and the loss of water-soluble FAAs during thawing. Fast freezing can effectively decrease the thawing loss of large yellow croaker, resulting in the highest value of total FAAs (112.00 mg/100 g) in TUIF samples. The most enriched FAAs in fresh and frozen samples were alanine, followed by lysine and glycine (Table 2). Aspartic acid, glutamic acid, alanine, and glycine provide the umami-sweetness taste in aquatic products. The UIF samples had higher alanine contents than the AF and IF samples, and TUIF samples showed the highest alanine content (20.35 mg/100 g). When the frequency is lower than 40 kHz, the cavitation intensity gets stronger with the increase in the frequency. Therefore, triple-frequency ultrasound can produce greater cavitation intensity than single-frequency and dual-frequency ultrasound (53). In the present study, histidine accounted for 2.61–4.04 mg/100 g of the total FAAs among all samples (Table 2). A low histidine content of (<5 mg/100 g) in large yellow croaker was recorded, which agreed with the results reported by Dou et al. (54) for large yellow croaker. Furthermore, the histidine content in large yellow croaker was much lower than that in other fish, such as silver carp (36.5 mg/100 g) (55) and grass carp (178.4 mg/100 g) (27). In summary, the results mentioned above indicated that flavour deterioration was associated with the decrease in special flavour-enhancing amino acids and the accumulation of flavour-detracting amino acids, and UIF treatment effectively delayed this process and maintained the good edible value of large yellow croaker during freezing.

**Table 2:** Changes in free amino acids (FAAs) content (mg/100 g) of large yellow croaker muscles after different freezing treatments.

**Samples**	**FAAs**								
	**Asp**	**Thr**	**Ser**	**Glu**	**Gly**	**Ala**	**Val**	**Met**	
Fresh	0.83 ± 0.01^e^	2.63 ± 0.04^f^	3.65 ± 0.05^f^	6.67 ± 0.11^d^	7.69 ± 0.11^f^	20.74 ± 0.22^b^	6.58 ± 0.07^a^	3.21 ± 0.02^a^	
AF	0.99 ± 0.01^d^	3.07 ± 0.04^e^	4.90 ± 0.06^c^	7.19 ± 0.10^c^	12.93 ± 0.06^b^	18.17 ± 0.16^d^	5.61 ± 0.06^c^	2.89 ± 0.04^b^	
IF	1.02 ± 0.03^d^	3.39 ± 0.00^d^	5.65 ± 0.02^a^	5.97 ± 0.05^e^	15.37 ± 0.02^a^	18.63 ± 0.04^c^	5.43 ± 0.01^d^	3.40 ± 0.02^a^	
SUIF	1.63 ± 0.02^b^	3.73 ± 0.06^c^	4.71 ± 0.08^d^	5.61 ± 0.02^f^	11.87 ± 0.19^c^	20.36 ± 0.30^b^	4.57 ± 0.06^f^	2.27 ± 0.04^d^	
DUIF	1.68 ± 0.03^a^	4.19 ± 0.03^a^	4.55 ± 0.01^e^	11.39 ± 0.04^a^	8.98 ± 0.04^e^	22.06 ± 0.08^a^	5.18 ± 0.05^e^	2.61 ± 0.07^c^	
TUIF	1.34 ± 0.01^c^	3.90 ± 0.04^b^	5.20 ± 0.02^b^	8.44 ± 0.09^b^	9.30 ± 0.02^d^	20.35 ± 0.08^b^	6.20 ± 0.04^b^	3.21 ± 0.18^a^	
	**Ile**	**Leu**	**Tyr**	**Phe**	**Lys**	**His**	**Arg**	**Pro**	**Total**
Fresh	4.50 ± 0.05^a^	7.63 ± 0.10^b^	3.45 ± 0.10^a^	3.68 ± 0.10^a^	14.41 ± 0.23^d^	2.60 ± 0.04^d^	0.01 ± 0.01^c^	2.97 ± 0.11^a^	91.26 ± 1.15^de^
AF	3.84 ± 0.04^b^	6.57 ± 0.07^c^	2.76 ± 0.05^d^	2.93 ± 0.03^c^	12.59 ± 0.23^e^	2.34 ± 0.04^e^	0 ± 0^c^	2.81 ± 2.12^a^	89.60 ± 1.37^e^
IF	3.76 ± 0.00^c^	6.33 ± 0.02^d^	3.34 ± 0.02^a^	3.18 ± 0.01^b^	20.81 ± 0.08^c^	3.97 ± 0.02^b^	0.03 ± 0.00^c^	2.55 ± 0.85^a^	102.81 ± 0.62^b^
SUIF	2.97 ± 0.03^d^	5.12 ± 0.07^e^	2.11 ± 0.03^e^	2.55 ± 0.04^d^	22.44 ± 0.33^b^	3.65 ± 0.05^c^	0.21 ± 0.01^a^	1.73 ± 0.11^a^	94.93 ± 1.15^c^
DUIF	3.82 ± 0.04^bc^	6.37 ± 0.08^d^	2.91 ± 0.08^c^	2.88 ± 0.17^c^	9.87 ± 0.17^f^	2.62 ± 0.05^d^	0.05 ± 0.00^b^	3.14 ± 0.05^a^	92.28 ± 0.77^d^
TUIF	4.54 ± 0.02^a^	8.12 ± 0.03^a^	3.10 ± 0.03^b^	3.00 ± 0.03^bc^	28.57 ± 0.20^a^	4.53 ± 0.04^a^	0.21 ± 0.00^a^	2.99 ± 0.03^a^	112.00 ± 0.79^a^

*Fresh, fresh fish meat; AF, air freezing; IF, immersion freezing; SUIF, single-ultrasound assisted freezing; DUIF, dual-ultrasound assisted freezing; TUIF, triple-ultrasound assisted freezing. The means in the same column with different letters differ significantly (p < 0.05)*.

### MP Primary Structure

#### Changes in Total Sulfhydryl Content

Changes in sulfhydryl content can indicate changes in protein folding, disulfide bond formation, and protein conformation (56). All samples had a decreased total sulfhydryl content compared with the fresh sample (Figure 3B). Oxidation of proteins can result in the creation of disulfide bonds due to the oxidation of some reactive sulfhydryl groups during freezing, thereby leading to a decrease in the total sulfhydryl group content (57). The total sulfhydryl group content gradually increased in the UIF samples with increasing ultrasound frequency, and the highest total sulfhydryl group content (85.09 μmol/g) was found in the TUIF sample. Zou et al. (58) found that the sulfhydryl content of ultrasonically treated chicken plasma protein increased significantly (*p* < 0.05) compared with that of non-treated samples. The increase in total sulfhydryl groups may presume that the cavitation effect generated from ultrasonic treatment can disrupt disulfide bonds during processing as a result of the interaction of microstreaming, shear forces, and turbulence (59). However, Sun et al. (16) reported that UAF at different power levels showed a significant difference (*p* > 0.05) in total sulfhydryl content, probably a result of the short freezing time.

#### Changes in Carbonyl Contents

The carbonyl content in fresh large yellow croaker was 2.34 nmol/mg (Figure 3C), and all samples showed an increase after freezing treatment. The UAF samples had lower carbonyl contents than the AF and IF samples, and TUIF had the lowest carbonyl content (2.59 nmol/mg). Amino acids, such as lysine, proline, and arginine, are prone to be oxidised to a semialdehyde in the presence of metal ions and reactive oxygen species (ROS), accounting for ~70% of the total quantity of carbonyl groups. Due to the cavitation effect, ultrasound treatment stimulates chemical responses resulting in the production of highly reactive free radicals, which can cause the thermal decomposition of water molecules into·OH,·H, and hydrogen peroxide (60). Additionally, lipid peroxides (carbonyl compounds and malondialdehyde) probably interact with proteins, generating carbonyl groups. This could be an explanation for the increase in the carbonyl concentration among all samples following the freezing process. However, the myofibril oxidation values of the UAF samples were clearly lower, demonstrating that the utilisation of ultrasound can effectually inhibit the myofibril oxidation of large yellow croaker samples. Sun et al. (16) also reported that the carbonyl content was notably higher (*p* < 0.05) in the high ultrasound power (UIF-200 and UIF-225) group than in the control group (*p* < 0.05). However, Shi et al. (60) stated that there were no significant changes (*p* > 0.05) in the carbonyl content in UAF grass carp samples.

### MP Secondary Structure

Fourier transform infrared spectroscopy is a widely applied means for evaluating the secondary structure of MPs, including α-helices, β-turns, β-sheets, and random coils (61). The amide I region (1,700 to 1,600 cm^−1^) is commonly applied for estimating MP secondary structure (Figure 3D). The fresh large yellow croaker samples contained 48.71% α-helices, 24.87% β-sheets, 15.61% β-turns, and 10.81% random coils (Figure 3E). The second-derivative-fitted curve of the amide I band of MP is shown in Figure 3F. All samples exhibited a decline in α-helical contents and a rise in β-sheet contents after the freezing treatments compared with the fresh sample. The content of α-helices in the UIF samples was higher than that in the AF (39.22 %) and IF (41.21 %) samples, and TUIF had the highest proportion of α-helices (43.30 %). The cavitation bubbles and microstreaming effects generated by trifrequency ultrasonic treatment accelerated the freezing process, resulting in the development of fine and well-distributed ice crystals, which decreased the damage to the fish muscles caused by the ice crystals. Stathopulos et al. (62) stated that ultrasonic treatment induced a decline in α-helix content together with a rise in the β-sheet structure of MPs. In contrast, Chandrapala et al. (63) found that ultrasound treatment (20 kHz, 450 W) resulted in a 10% gain in the α-helix component and a 6–9% decrease in the β-sheet contents of whey protein concentrate. The FTIR results in this research indicated that freezing and ultrasonic treatment both affected the MP secondary structure, and that multifrequency ultrasound (especially TUIF) can decrease the changes in MP secondary structure resulting from freezing.

### MP Tertiary Structure

Intrinsic fluorescence intensity reflects the variations in MP tertiary structure (64). In general, tryptophan is in a non-polar environment if the λ_max_ is lower than 330 nm, whereas tryptophan is in a polar environment if the λ_max_ is higher than 330 nm (65). In this study, the fresh large yellow croaker samples revealed a λ_max_ value of 336.2 nm (Figure 3G), suggesting that tryptophan residues were in a polar atmosphere. The freezing process resulted in a transition of λ_max_ to longer wavelengths, ranging from 336.2 nm (fresh) to 337.3 nm (AF), demonstrating that parts of the buried tryptophan residues were exposed to a polar environment. These variations may be due to the different energy transfer efficiencies between tryptophan and tyrosine or exposure of the sample chromophore to solvent following freezing (66). No significant differences were observed in the λ_max_ values in samples (*p* > 0.05). The samples with UIF treatment had lower λ_max_ values than the samples with AF and IF treatment, which may be due to the larger ice crystals induced by AF and IF, which destroyed the muscle properties. Zhang et al. (67) pointed out that samples that underwent freeze-thaw treatment exhibited an increased FI_max_ compared to fresh samples, which might be due to protein–protein association induced by the freezing-thawing process. However, Wang et al. (22) stated that the IFI of MPs with different thawing methods was lower than that of fresh samples because of the unfolding of the MPs during rapid freezing and thawing. The IFI of the TUIF samples was significantly (*p* < 0.05) larger than those of the DUIF and SUIF samples. This is probably because triple-frequency ultrasound has a significant effect on the MP structure by producing resonance with its own frequencies of MP. The resonance can cause a stronger effect on the MP samples, and thus the tertiary structure of MP was improved (44). Thus, multifrequency ultrasonic treatment accelerated freezing and reduced the destruction of muscle properties caused by ice crystals (38). The outcomes were further validated by microstructure observations, suggesting that multifrequency ultrasonic treatment can decrease injury to frozen fish tissue.

## Conclusions

This study investigated the influence of multifrequency ultrasonic treatments on the flavour attributes and MP characteristics of large yellow croaker. Multifrequency ultrasound slowed the accumulation of TVB-N in large yellow croaker during freezing process. The SEM images showed that multifrequency ultrasound contributed to the generation of finer and more uniform ice crystals to reduce muscle tissue damage. These effects become more obvious as the ultrasonic frequency increased. The quality of the samples frozen by TUIF was found to be better than that of the samples frozen by SUIF and DUIF. The formation of undesirable volatile flavour compounds was also inhibited by multifrequency ultrasonic treatment. The structure of multifrequency ultrasound samples was more stable than that of the AF and IF samples according to the FTIR and fluorescence spectra. These results indicated that multifrequency ultrasonic treatment efficiently improved the flavour attributes and MP characteristics of large yellow croaker. Therefore, multifrequency UAF is a promising food engineering technology to build sustainable and resilient food systems, improve nutritional profile of foods, and increase food and nutrition security.

## Data Availability Statement

The raw data supporting the conclusions of this article will be made available by the authors, without undue reservation.

## Ethics Statement

Ethical review and approval was not required for the animal study because this study did not involve in live animal experiments.

## Author Contributions

XM and JM: conceptualization and writing the original draft preparation. DY and WQ: methodology. XM and WQ: data curation. JM and JX: writing, reviewing, and editing. DY and JX: funding acquisition. All authors contributed to the article and approved the submitted version.

## Funding

This research was funded by the National Key R&D Program of China (2019YFD0901603).

## Conflict of Interest

The authors declare that the research was conducted in the absence of any commercial or financial relationships that could be construed as a potential conflict of interest.

## Publisher's Note

All claims expressed in this article are solely those of the authors and do not necessarily represent those of their affiliated organizations, or those of the publisher, the editors and the reviewers. Any product that may be evaluated in this article, or claim that may be made by its manufacturer, is not guaranteed or endorsed by the publisher.
